# Protection against T1DM-Induced Bone Loss by Zinc Supplementation: Biomechanical, Histomorphometric, and Molecular Analyses in STZ-Induced Diabetic Rats

**DOI:** 10.1371/journal.pone.0125349

**Published:** 2015-05-01

**Authors:** Raul Hernandes Bortolin, Bento João da Graça Azevedo Abreu, Marcela Abbott Galvão Ururahy, Karla Simone Costa de Souza, João Felipe Bezerra, Melina Bezerra Loureiro, Flávio Santos da Silva, Dáfiny Emanuele da Silva Marques, Angélica Amanda de Sousa Batista, Gisele Oliveira, André Ducati Luchessi, Valéria Morgiana Gualberto Duarte Moreira Lima, Carlos Eduardo Saraiva Miranda, Marcus Vinicius Lia Fook, Maria das Graças Almeida, Luciana Augusto de Rezende, Adriana Augusto de Rezende

**Affiliations:** 1 Department of Clinical and Toxicological Analyses, Federal University of Rio Grande do Norte, Natal, Rio Grande do Norte, Brazil; 2 Department of Morphology, Federal University of Rio Grande do Norte, Natal, Rio Grande do Norte, Brazil; 3 Department of Pharmacy, State University of Paraiba, Campina Grande, Paraiba, Brazil; 4 Department of Chemistry, University of Ribeirão Preto, Ribeirão Preto, São Paulo, Brazil; 5 Department of Pharmacy, University of Ribeirão Preto, Ribeirão Preto, São Paulo, Brazil; 6 Laboratory of Evaluation and Development of Biomaterials, Federal University of Campina Grande, Campina Grande, Paraiba, Brazil; Faculté de médecine de Nantes, FRANCE

## Abstract

Several studies have established an association between diabetes and alterations in bone metabolism; however, the underlying mechanism is not well established. Although zinc is recognized as a potential preventive agent against diabetes-induced bone loss, there is no evidence demonstrating its effect in chronic diabetic conditions. This study evaluated the effects of zinc supplementation in a chronic (90 days) type 1 diabetes-induced bone-loss model. Male Wistar rats were distributed in three groups: control, type 1 diabetes mellitus (T1DM), and T1DM plus zinc supplementation (T1DMS). Serum biochemical analysis; tibia histomorphometric, biomechanical, and collagen-content analyses; and femur mRNA expression were evaluated. Relative to T1DM, the zinc-supplemented group showed increased histomorphometric parameters such as TbWi and BAr and decreased TbSp, increased biomechanical parameters (maximum load, stiffness, ultimate strain, and Young’s modulus), and increased type I collagen content. Interestingly, similar values for these parameters were observed between the T1DMS and control groups. These results demonstrate the protective effect of zinc on the maintenance of bone strength and flexibility. In addition, downregulation of *OPG*, *COL1A*, and *MMP-9* genes was observed in T1DMS, and the anabolic effects of zinc were evidenced by increased OC expression and serum ALP activity, both related to osteoblastogenesis, demonstrating a positive effect on bone formation. In contrast, T1DM showed excessive bone loss, observed through reduced histomorphometric and biomechanical parameters, characterizing diabetes-associated bone loss. The bone loss was also observed through upregulation of *OPG*, *COL1A*, and *MMP-9* genes. In conclusion, zinc showed a positive effect on the maintenance of bone architecture and biomechanical parameters. Indeed, *OC* upregulation and control of expression of *OPG*, *COL1A*, and *MMP-9* mRNAs, even in chronic hyperglycemia, support an anabolic and protective effect of zinc under chronic diabetic conditions. Furthermore, these results indicate that zinc supplementation could act as a complementary therapy in chronic T1DM.

## Introduction

Type 1 diabetes mellitus (T1DM) is a chronic disease in which pancreatic beta cells are selectively destroyed, leading to chronic hyperglycemia [1], and several consequential long-term vascular complications such as retinopathy, neuropathy, and nephropathy have been reported [1–3]. Although skeletal abnormalities and bone disease represent an overlooked complication of diabetes, the relationship is well established and recognized as a complex pathogenesis including mechanical, hormonal, and vascular factors involving an imbalance between bone formation and resorption.

Diabetes-induced bone-loss mechanisms are not fully understood [4–7] although a variety of bone-related changes are known to be influenced by hyperglycemia such as bone mineral density, femoral neck geometry, microarchitecture (trabecular, cortical thickness, and bone area), and biomechanical markers of bone turnover (ultimate strain, strength and load, stiffness, and Young’s modulus) [3,8–11]. In addition, formation of the collagen network is affected by an increase in matrix metalloproteinase (MMP) expression in diabetic conditions, especially for MMP-9, which is considered a diabetogenic factor and is upregulated in T1DM, contributing to collagen degradation and resulting in low bone collagen content and poor bone biomechanical integrity [12–15]. One study showed a decrease in bone mineral content (BMC) associated with an increase in urinary calcium excretion in diabetic rats. Moreover, low BMC in diabetic conditions has been associated with low osteocalcin (OC) levels, as this small non-collagenous protein is produced by osteoblasts and is directly involved in bone inorganic matrix development [16].

Several studies have reported controversial alterations in the *RANK/RANKL/OPG* (receptor activator of nuclear factor kappa β/receptor activator of nuclear factor kappa β ligand/osteoprotegerin) system in hyperglycemic conditions [2,4,8,17–28]. Some studies have shown an increase in *RANKL* mRNA expression in diabetic bone [2] and decreases in a high-glucose *in vitro* model [18] and diabetic bone [20]. *OPG* expression has been shown to increase in young T1DM patients [8], whereas a decrease in gene expression was also observed in T1DM patients [4], in a T1DM animal model [19], and in an *in vitro* study [17].

As an alternative, investigators have used zinc supplementation for bone-loss prevention in both healthy and hyperglycemic conditions because it is an essential element in bone metabolism, acting as a cofactor for several enzymes and stimulating gene expression of various proteins necessary for bone mineralization and collagenous structure development [10,21–25].

Studies involving *in vitro* and *in vivo* models have evaluated the efficacy of zinc supplementation in preventing bone loss [22,23,26–28]. *In vitro* results have shown a stimulatory effect on osteoblastogenesis through increases in DNA, collagen, calcium, insulin-like growth factor 1 (IGF-I), transforming growth factor beta 1 (TGF-β1), alkaline phosphatase (ALP) activity, and OC [21–22]. Furthermore, an *in vivo* study involving acute T1DM-induced bone loss and zinc supplementation showed a significant effect of zinc on bone formation associated with an increase in *OC* mRNA expression [23].

A positive effect of zinc on the recovery of bone architecture has been reported in acute diabetic conditions [23], and bone biomechanical tests in rats showed that zinc maintained overall bone quality and increased fracture resistance [24–25]. Moreover, the effects of zinc on the *RANK/RANKL/OPG* system has been reported by Yamaguchi [22], who demonstrated the inhibition of *RANKL* expression in pre-osteoclasts and the stimulation of *OPG* gene expression in osteoblastic cells, which could act as a decoy receptor by binding to RANKL and preventing RANK signaling.

Although a few reports have shown positive effects of zinc supplementation, all of these studies were performed during an acute period between 7 and 21 days [23,29]. Our aim was to evaluate the effect of zinc under long-term diabetic conditions to provide proof-of-concept for the potential use of zinc supplementation in preventing chronic T1DM-induced bone loss. To the best of our knowledge, this is the first study to evaluate the effects of zinc supplementation over a period of 90 days, which represents a chronic model of diabetes, and provide evidence that zinc ingested as dietary supplement can prevent bone loss through anabolic and osteo-protective effects. We show that this *in vivo* action results from the stimulation of bone formation and decreased bone resorption as detected by histomorphometric, collagen-content, biomechanical, and quantitative reverse transcription PCR (RT-qPCR) analyses. Together with prior evidence showing a relationship between supplementation and bone metabolism, our data provide compelling evidence for the therapeutic potential of zinc supplementation as a complementary therapy against chronic T1DM-induced bone loss.

## Methods

### Experimental Protocol

All animal experiments and protocols were approved by the Committee on the Ethics of Animal Use and Care of the Federal University of Rio Grande do Norte (permit number 022/2009) and the Committee on the Ethics in Research of the University of Ribeirão Preto (permit number 066/09). All procedures were carried out in strict accordance with the recommendations in the Guide for the Care and Use of Laboratory Animals of the National Institutes of Health [30]. All surgery was performed under thiopental anesthesia, and all efforts were made to minimize suffering.

Fifteen male Wistar rats weighing 220 ± 20 g were obtained from the Laboratory Animal Facility of the University of Ribeirão Preto, Ribeirão Preto, Brazil. During the study period, the animals were housed in standard conditions (12 h light/dark cycle, 22–24°C, and 50–60% humidity) with food and water *ad libitum*. After one week of acclimatization prior to the experimental procedures, the rats were randomly assigned and equally distributed (five rats per group) to three groups: control, T1DM, and T1DM plus zinc supplementation (T1DMS).

Experimental diabetes was induced by a single intravenous injection of streptozotocin (STZ, Sigma-Aldrich, St. Louis, MO, USA) dissolved in freshly prepared Na citrate buffer (0.1 M, pH 4.5) at a dose of 40 mg/kg of body weight. Equal volumes of vehicle were injected in the control rats. On day 0, i.e., day 5 after induction, blood samples were collected by tail bleeding, and glycemia was assayed using an ACCU-CHEK Advantage glucometer (Roche Diagnostics, Indianapolis, IN, USA). Animals with blood glucose concentrations ≥250 mg/dL were considered diabetic and started receiving a standard (control and T1DM) or supplemented diet (T1DMS). The blood glucose concentrations and body weight were monitored fortnightly for 12 weeks. Clinical diabetic signs such as polyphagia, polydipsia, polyuria, and body weight loss were also monitored [31,32].

Standard diets were formulated in accordance with rodent-specific rules established by the American Institute of Nutrition in 1993 (AIN-93) [33]. Dietary ingredients were provided by Rhoster Industry and Trade Ltd. (São Paulo, Brazil). The control and T1DM groups were fed daily with 20 g of standard diet for 12 weeks. Because the AIN-93 standard diet contains 30 mg zinc/kg diet, we supplemented the diet with 500 mg zinc/kg diet; thus, the T1DMS group was fed a standard diet supplemented with 17-fold geometrically diluted ZnCO_3_. All animals in the T1DMS group consumed 20 g of the supplemented diet daily during the 12 weeks of study, totaling 10.6 mg ZnCO_3_ ingestion daily.

### Blood and tissue collection and routine biochemical analyses

All animals were euthanized by a lethal dose of thiopental (100 mg/kg), and blood samples were obtained from the abdominal aorta. To prevent possible daily cyclic variations of the measurements, all animals were euthanized between 7:00 am and 9:00 am. The femurs and tibias were harvested and stored for subsequent total RNA extraction and histomorphometric, biomechanical, and collagen-content analyses.

Serum glucose concentration and ALP activity were determined in triplicate using routine methods (BioSystems Reagents and Instruments, Barcelona, Spain) and performed in an RA 50 spectrophotometer (Chemistry System Bayer Diagnostic, Dublin, Ireland). Serum zinc was measured by atomic absorption spectroscopy using a Spectra AA-200 spectrophotometer (Varian Canada, Georgetown, Ontario, Canada).

### Histomorphometric analyses

The right tibia of each animal was fixed in a solution of 10% buffered formalin and processed after decalcification in 7.5% nitric acid and embedding in paraffin following standard procedures as described by Duarte et al. [34] with modifications. Longitudinal 7-μm sections were stained with hematoxylin and eosin. The histomorphometric results are presented as the means of four measurements of trabecular separation (TbSp, μm), trabecular width (TbWi, μm) and trabecular bone area (BAr, %) obtained from the metadiaphyseal region using a Nikon Lobophot microscope equipped with a 10× magnification ocular lens (Nikon, Tokyo, Japan). After analysis using ImageJ 1.48v (National Institutes of Health, Bethesda, MD, USA), the results were reported in μm. All parameters complied with the guidelines of the Nomenclature Committee of the American Society of Bone and Mineral Research [35].

### Collagen content

Paraffin-embedded samples were also used for collagen quantification by picrosirius red staining according to the procedure described by Rich and Whittaker [36] with modifications. To evaluate the collagen content in sections stained with picrosirius red, four fields of the metadiaphyseal region were observed with a 10× magnification ocular lens in an AxioImager M2 microscope (Carl Zeiss, Jena, Germany). In each field, the percentages of tissue area stained in red and green relative to the total tissue area were calculated according to the formula described by Black et al. [37]. All analyses were performed using ImageJ 1.48v.

### Biomechanical testing

Biomechanical analysis was performed on the left tibias previously stored at −80°C using three-point bending mechanical tests according to the procedure described by Korres et al. [12], with modifications. We used a servo hydraulic high-precision universal testing machine, model AG-X 10 kN (Shimadzu Corporation, Tokyo, Japan). Tibias were placed horizontally on the frame with rounded edges at a distance of 30 mm. The load was applied at the mid-shaft of the diaphysis using a punch with a rounded notch. The rate of the imposed displacement was selected as 5 mm/min to simulate static loading conditions. The displacement was imposed continuously until fracture. Failure in the load-displacement curves was defined and observed by the propagation of a nearly vertical fracture starting almost universally at the lower cortical bone surface. Ultimate load, stiffness, ultimate stress, ultimate strain, and Young’s modulus were recorded.

### RNA extraction and RT-qPCR

The right femurs of the animals, previously stored at −80°C, were pulverized, and total RNA was extracted using an RNeasy Plus Mini Kit (Qiagen, Valencia, CA, USA). The RNA integrity was assessed by electrophoresis in 1.0% agarose gels with MOPS buffer, concentrations were measured using a Nanodrop ND-1000 spectrophotometer (Thermo Scientific, Wilmington, DE, USA), and RNA was stored at −80°C. Synthesis of cDNA was performed with 1 μg total RNA using a High Capacity cDNA Reverse Transcription Kit (Applied Biosystems, Foster City, CA, USA), according to the manufacturer’s protocol in a MyCycler Thermal Cycler (Bio-Rad, Philadelphia, PA, USA). The cDNA was obtained in a final volume of 50 μL and stored at −20°C until it was used for the RT-qPCR expression assays.

RT-qPCR was performed on the following genes using the TaqMan Assay: *RANKL* (Rn00589289_m1), *OPG* (Rn00563499_m1), *OC* (Rn00566386_g1), *COL1A1* (Rn01463848_m1), *MMP-2* (Rn01538170_m1), *MMP-9* (Rn00579162_m1), and glyceraldehyde-3-phosphate dehydrogenase (*GAPDH*, Rn00579162_m1) (Applied Biosystems). PCR assays were carried out in 96-well plates using a 7500 Fast Real-time PCR System (Applied Biosystems). Relative expression was calculated using the 2-ΔΔCT method [38], and results are presented as fold-change versus the control group mean values, normalized to *GAPDH*; Ct did not show significant variation between the control and T1DM groups.

### Statistical analyses

Statistical analyses were performed with GraphPad PRISM version 5.0 (GraphPad Software Inc., San Diego, CA, USA). In all data, the normality test failed, and we therefore used the nonparametric Kruskal-Wallis ANOVA on Ranks and Dunn’s post-hoc method of multiple comparisons versus control. *p*-values < 0.05 were considered statistically significant.

## Results

### Biochemical analyses and body weight

Biochemical analyses and body weight results are shown in Table 1. As expected, blood glucose concentrations in T1DM and T1DMS rats were than those in control rats (*p* < 0.001). Hyperglycemia was associated with polyphagia, polydipsia, and polyuria (data not shown) in the diabetic rats, indicating that experimental diabetes was successfully induced. The baseline body weight at the beginning of the study was similar in control and diabetic groups (average 220 ± 20 g). However, after 90 days of the experimental period, which represents a chronic condition, T1DM and T1DMS groups showed significantly reduced body weight (*p* < 0.001).

**Table 1:** Biochemical analyses and body weight of control, diabetic, and diabetic plus zinc supplementation groups.

	Control	T1DM	T1DMS
Body Weight(g)	336.00±20.74	153.40±76.51*^/###^	157±14.44*^/###^
Glucose(mg/dL)	97.40±18.96	598.40±76.51*^/###^	604.8±165.80*^/###^
ALP(U/L)	174.80±126.1	346.20±208.40	1803±474.70*^/###^
Zinc(μg mL^−1^)	1.61±0.3889	0.79±0.33*^/#^	2.71±0.47*^/##^ **^/##^

T1DM, type 1 diabetes mellitus; T1DMS, T1DM plus zinc supplementation; ALP, alkaline phosphatase. All data are shown as means ± SEM. Comparisons between groups were analyzed with Kruskal-Wallis ANOVA on Ranks and Dunn’s post-hoc.

*^/#^ *p* < 0.05 vs. control group;

*^/##^ *p* < 0.01 vs. control group;

*^/###^ *p* < 0.001 vs. control group;

**^/##^ *p* < 0.01 vs. T1DM group.

No significant difference was observed in serum ALP activity between the T1DM and control groups. However, ALP activity was significantly higher in the T1DMS group than in the control and T1DM groups (*p* < 0.001).

Serum zinc concentration was decreased in T1DM rats compared to control rats (*p* < 0.05), and we observed the expected increase in serum zinc concentration in T1DMS rats compared to the control and T1DM groups (*p* < 0.01).

### Histological and histomorphometric analyses

**Fig 1:**
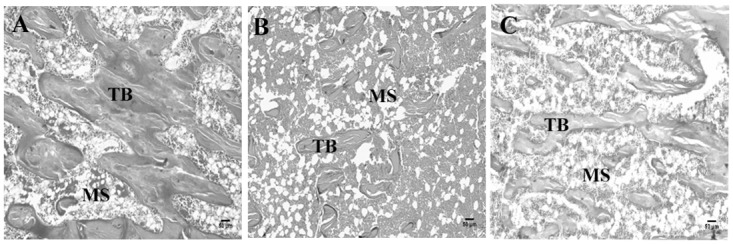
Histological analyses of the right tibias. Hematoxylin and eosin staining of longitudinal sections of tibias of the control (A), type 1 diabetes mellitus (T1DM) (B), and T1DM plus zinc supplementation (T1DMS) (C) groups. TB, trabecular bone; MS, medullary space; magnification 20X, scale bar: 50 μm.

**Fig 2:**
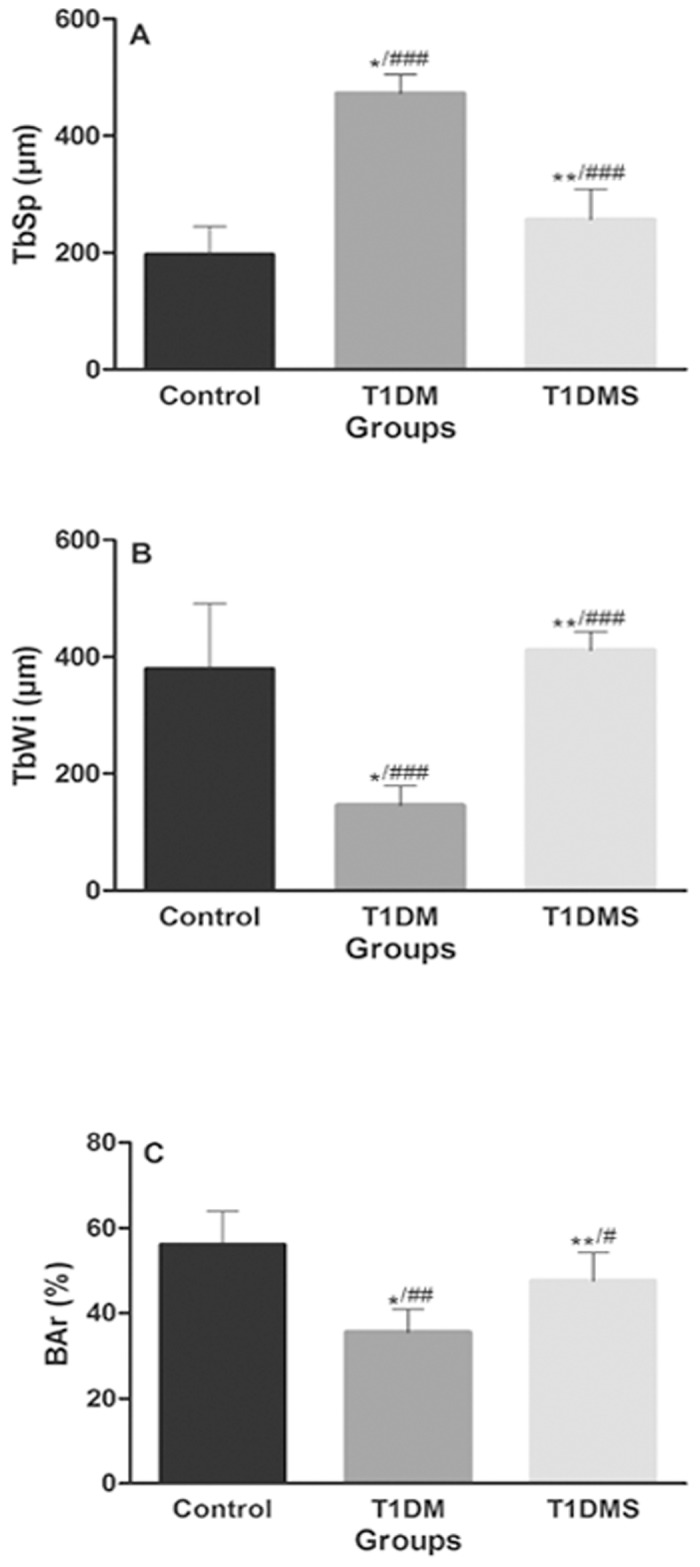
Histomorphometric analyses of structural bone architecture. Trabecular separation (TbSP, μm) (A), trabecular width (TbWi, μm) (B), and trabecular bone area (BAr, %) (C) of control, type 1 diabetes mellitus (T1DM), and T1DM plus zinc supplementation (T1DMS) rats. All data are shown as means ± SEM. Comparisons between groups were analyzed with Kruskal-Wallis ANOVA on Ranks and Dunn’s post-hoc. *p* < 0.01*^/##^ vs. control group; *p* < 0.001*^/###^ vs. control group; *p* < 0.05 **^/#^ vs. T1DM group; *p* < 0.001**^/###^ vs. T1DM group.

Histological and histomorphometric results are shown in Figs 1 and 2, respectively. Increased TbSp (Fig 2A) and decreased TbWi (Fig 2B) and BAr (Fig 2C) were observed in the T1DM group compared to the control group (*p* < 0.001, *p* < 0.001, and *p* < 0.01, respectively). The T1DMS group showed reduced TbSp and increased TbWi and BAr compared to the T1DM group (Fig 1, *p* < 0.001, *p* < 0.001, and *p* < 0.05, respectively). In addition, T1DMS rats exhibited similar results when compared to control rats. Representative histological data are shown in Fig 1.

### Collagen content

**Fig 3:**
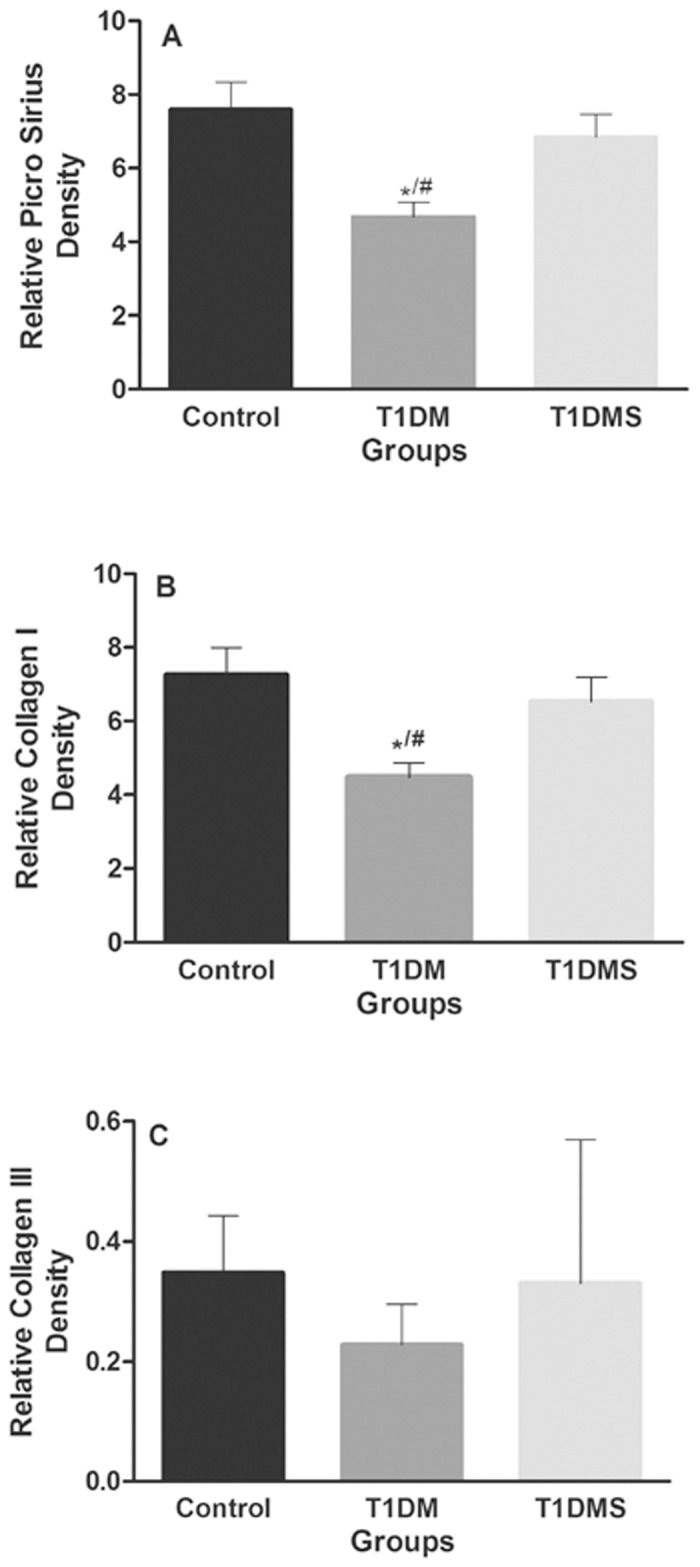
Assessment of collagen deposition by picrosirius red staining. Tibia staining for collagen content (picrosirius red). Total collagen (A), collagen type I (B), and collagen type III (C) contents of the control, type 1 diabetes mellitus (T1DM), and T1DM plus zinc supplementation (T1DMS) groups. All data are shown as means ± SEM. Comparisons between groups were analyzed with Kruskal-Wallis ANOVA on Ranks and Dunn’s post-hoc. *p* < 0.05 *^/#^ vs. control group.

The collagen analysis results are shown in Fig 3. We observed a decrease in total collagen in T1DM rats compared to control rats (Fig 3A, *p* < 0.05), whereas there was no difference between T1DMS rats and control rats. We also found a decrease in type I collagen in T1DM rats compared to controls (Fig 3B, *p* < 0.05), and T1DMS results were increased 1.2-fold compared to the T1DM group; however, this increase was not statistically significant. No differences were observed for type III collagen (Fig 3C).

### Biomechanical testing data

Biomechanical parameters are shown in Table 2. Significantly decreased values for ultimate load, stiffness, ultimate strain, and Young’s modulus in the T1DM group was observed relative to control values (*p* < 0.01, *p* < 0.01, *p* < 0.05, and *p* < 0.05, respectively). Interestingly, the values of these parameters were higher (2-, 1.5-, 1.2-, and 1.2-fold, respectively) in the T1DMS group than in the T1DM group. In addition, we observed that ultimate load, ultimate strain, and Young’s modulus values in the T1DMS group were similar to the control values. The stiffness was uniquely decreased in T1DMS rats compared to controls (*p* < 0.05) but showed a 1.2-fold increase compared to T1DM rats. No significant difference was observed in the ultimate stress parameter.

**Table 2:** Tibia biomechanical parameters of control, diabetic, and diabetic plus zinc supplementation groups.

Property/Groups	Control	T1DM	T1DMS
Ultimate load(N)	82.85±8.39	48.42±14.71*^/##^	66.80±9.04
Stiffness(N/mm)	133.21±4.21	86.95±16.80*^/##^	102.21±6.75*^/#^
Ultimate Stress(N/mm^2^)	413.50±24.32	392.90±122.60	379.60±108.80
Ultimate Strain(%)	1.67±0.24	1.23±0.26*^/#^	1.52±0.18
Young´s modulus(GPa)	24.04±1.58	20.07±1.83*^/#^	23.37±3.21

T1DM, type 1 diabetes mellitus; T1DMS, T1DM plus zinc supplementation. All data are shown as means ± SEM. Comparisons between groups were analyzed with Kruskal-Wallis ANOVA on Ranks and Dunn’s post-hoc.

*^/#^ *p* < 0.05 vs. control group;

*^/##^ *p* < 0.01 vs. control group.

### mRNA expression data

**Fig 4:**
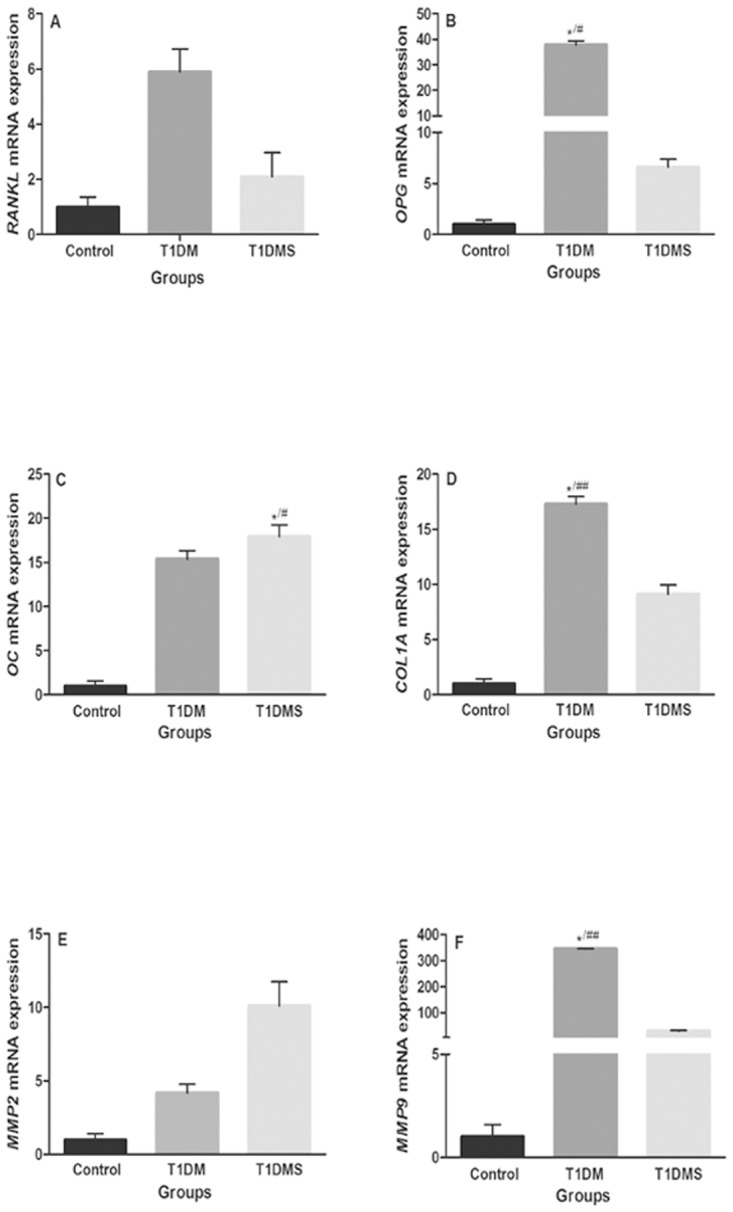
Relative mRNA expression quantification. *RANKL* (A), *OPG* (B), *OC* (C), *COL1A* (D), *MMP-2* (E), and *MMP-9* (F) mRNA expression in bone tissue of control, type 1 diabetes mellitus (T1DM), and T1DM plus zinc supplementation (T1DMS) rats. All data are expressed as fold-change vs. control group values, normalized to *GAPDH*. Comparisons between groups were analyzed with Kruskal-Wallis ANOVA on Ranks and Dunn’s post-hoc. *p* < 0.05*^/#^ vs. control group; *p* < 0.01*^/##^ vs. control group.

Molecular bone metabolic parameters are summarized in Fig 4. The mRNA expression levels of *OPG*, *COL1A*, and *MMP-9* (Fig 4B, 4D, and 4F, respectively) were increased 37.8-, 17.3-, and 344.4-fold, respectively (*p* < 0.05, *p* < 0.01, and *p* < 0.01, respectively) in T1DM rats compared with control rats. Interestingly, the T1DMS group showed decreased expression of these genes (31.1-, 8.3-, and 313.9-fold, respectively) in comparison to T1DM rats, although this result was not statistically significant. In addition, no alterations were observed in these genes between the control and T1DMS groups (Fig 4B, 4D and 4F) except the *OC* mRNA expression, which showed a 17.9-fold increase (*p* < 0.05) compared with the control group (Fig 4C). No significant difference was found for *MMP-2* mRNA expression between the groups.

## Discussion

Several studies have shown that bone turnover and skeletal integrity are affected by diabetes; however, the underlying mechanism of diabetes-induced bone loss remains elusive, as does the influence of disease stage in its development [3,4,17,34].

The STZ-induced diabetes model has been extensively used, making it particularly useful for building upon and comparing study results [2,10,23,29,37,39–42]. The benefits of the STZ-induced diabetic model include the ability to induce diabetes in a genetically altered animal, maintain the model in a controlled environment, regularly monitor and directly measure serum and bone factors, obtain bone samples for high-resolution analyses, and choose the time of diabetic induction (compared to waiting for diabetes to occur in spontaneous models) [2]. Indeed, STZ induction of diabetes causes a bone phenotype consistent with human studies [8,43–49] and with spontaneous mouse models such as NOD mice [50], confirming the utility of the STZ model for studying mechanisms of T1-diabetes-induced bone loss.

Several studies have used zinc supplementation to preserve bone structure and metabolism, as zinc is an essential nutrient for human and animal growth [22,25,26,51,52]. Zinc deficiency during adolescence may increase the risk of bone disease later in life due to reduced mineralization during the consolidation phase of bone mineral acquisition [53]. The protective effect of zinc on bone is suggested primarily by its stimulatory effect on cell proliferation, differentiation, and mineralization in osteoblasts, thereby promoting bone formation [21,22]. Additionally, zinc may stimulate the expression of various cellular proteins, including Runx2/Cbfa1 (Runt-related transcription factor 2/Core binding factor alpha 1), type I collagen, ALP, and OC. Zinc also increases cellular production of IGF-I and TGF-β1. Moreover, this ion also suppresses the osteoclast-like cell formation induced by various bone-resorbing factors in bone marrow culture (e.g., RANKL in pre-osteoclasts) and stimulates *OPG* gene expression in osteoblastic cells, which can inhibit the binding of RANKL to RANK in pre-osteoclastic cells [21].

Herein, we chose to evaluate the zinc protective effect for a period of 90 days of T1DM because chronic high glucose exposure may present an extreme condition for studying key aspects of bone alteration such as architecture, biomechanics, and gene regulation.

Although the beneficial influence of zinc is recognized in T1DM-related bone loss, few studies have evaluated the effect of zinc supplementation in chronic conditions [23,27,29,42,54,55]. The majority of studies have shown a zinc protective effect in short-term T1DM animal models, restoring calcium content, ALP activity, and DNA content after 14 days of T1DM onset [29,42]. Furthermore, zinc prevented diabetes-induced osteoclastogenesis and decreased osteoblastogenesis two weeks after diabetes onset, as evaluated by histomorphometric, biochemical, and molecular parameters [23].

Our results demonstrate significant bone loss associated with long-term T1DM and, because zinc supplementation was initiated following diabetes onset, suggest an important protective effect of zinc against excessive bone loss in chronic T1DM.

The protective effect of zinc was supported through histomorphometric parameters, which showed decreased TbSp and increased TbWi and BAr in T1DMS rats relative to T1DM rats. These results emphasize the importance of supplementation as an anabolic and protective agent against reduction in bone architecture. Similarly, in a T1DM-induced bone loss model over 14 days, Iitsuka et al. [23] showed recovery of BAr, TbWi, and the number of osteoclasts and osteoblasts in the supplemented group, suggesting that zinc restored diabetes-induced osteopenia by acting on both bone formation and resorption through regulation of osteoclast and osteoblast numbers. Furthermore, in normal rats, Ovesen et al. [24] found that alimentary zinc deficiency in growing rats reduced distal femoral metaphysis and femoral diaphysis during four weeks of study.

The protective effect of zinc supplementation was highlighted by altered trabecular structures (increased TbSp and diminished TbWi and BAr) in the T1DM group, evidencing negative effects of hyperglycemia on bone structure and leading to significant bone loss. These results corroborate a previous 120-day study in our laboratory [34] in which we observed a significant increase in TbSp, a decrease in TbWi, and a progressive ~77% reduction in BAr accompanied by a proportional expansion of the marrow space. Other investigators observed alterations in TbWi and TbSp two and eight weeks after the onset of experimental diabetes, respectively [39,56]. Moreover, our results are consistent with μ-CT analyses by Thrailkill et al. [3] and Martin and McCabe [57], even though these studies employed a short-term diabetes model.

In addition to histomorphometric analyses, the biomechanical integrity of bone is an important factor affecting the risk of fracture. Diabetes-induced structural abnormalities that predispose bone to fractures may occur spontaneously or with minimal trauma in patients [58].

In the present study, zinc supplementation prevented diabetes-induced alterations: the T1DMS group showed increased mineralization content (stiffness parameter) and bone strength (ultimate stress parameter), which may reflect the bone resistance to fracture, compared to the T1DM group, and values were similar between the T1DMS and control groups [59]. Furthermore, maintenance of the inorganic matrix may preserve the bone flexibility observed through increased ultimate strain and Young’s modulus in T1DMS rats compared to T1DM rats. Young’s modulus is a basic material property that is independent of geometry, represents the ability of bone to resist deformation, and is associated with ultimate strain in reflecting important parameters related to bone flexural conditions [59].

Only few studies have investigated the effect of zinc on bone biomechanical parameters, however in a non-diabetic rodent model [24,25]. To the best of our knowledge, the present study is the first to evaluate the effect of zinc supplementation on chronic T1DM-induced bone loss through bone biomechanical parameters.

The zinc protective effect was supported through reduced values for biomechanical parameters (stiffness, ultimate stress, ultimate strain, and Young’s modulus) in the T1DM group. These results suggest that the bone integrity changes observed under conditions of high glucose exposure may be attributed to interrelated factors such as macroscopic structure (size and shape), architecture (cortical and tissue), and bone substance (organic and inorganic components), all of which may influence mechanical strength.

Additionally, the reduction in biomechanical parameters in T1DM rats agrees with studies using similar tests at seven [11] and eight weeks after diabetes confirmation [10,12].

Interestingly, the increase in biomechanical parameters was associated with increased collagen content in the zinc supplementation group, further suggesting an important role for zinc in maintenance of the organic matrix during this long-term (90 days) study. However, for the T1DM group, the reduction in type I collagen content supports the low biomechanical properties found after 90 days of study.

Finally, we analyzed mRNA expression levels of key genes associated with bone metabolism. To the best of our knowledge, this is the first study to evaluate the effects of zinc supplementation in a chronic model of diabetes-induced bone loss (90 days) through the analysis of *RANKL*, *OPG*, *OC*, *COL1A*, *MMP-2*, and *MMP-9* mRNA expression.

*MMP-9* was downregulated in the T1DMS group relative to the T1DM group, indicating maintenance of bone type I collagen and correlating with the greater flexural strength observed in zinc-supplemented rats. On the other hand, the upregulation of *MMP-9* and the low biomechanical and histomorphometric properties of the T1DM group suggest bone loss under the hyperglycemic condition. This hypothesis is supported by the association between MMP-9 and degradation of bone collagens in the subosteoclastic microenvironment and its potential role in normal bone remodeling and pathologic bone resorption [60].

MMP-9, a proteolytic member of the metalloproteinase family also named 92-kD type IV collagenase (gelatinase B), can degrade the components of the bone organic matrix and process both helical and denatured forms of type I collagen [13,60]. It is expressed at high levels in rabbit and human osteoclasts and in multinucleated cells of giant-cell tumors of bone [14]. Okada et al. [61] reported that MMP-9 has relatively broad substrate specificity, hydrolyzing collagen types I, III, IV, and V, and gelatins, and 50–80% of its full activity is retained at acidic pH. Grigoriadis et al. [62] showed that the expression of this enzyme is altered by bone-resorption activity.

Studies in non-diabetic osteoporotic bone demonstrated that osteoclasts can synthesize MMP-9 and exocytose it to degrade bone matrix components, especially type I collagen. Interestingly, some cells lining the surface of the bone matrix also express MMP-9, which is indirectly involved in bone resorption by removing the osteoid layer in this context, thereby exposing mineralized bone matrix and facilitating the adhesion of osteoclasts [63]. Although the hyperexpression of MMP-9 may contribute to collagen degradation, an important role for MMP-9 in healthy bone was reported by Nyman et al. [13] using MMP-9^−^/^−^ mice, which showed changes in the trabecular architecture and cortical structure compared with wild-type mice, suggesting that bone quality is influenced by the MMPs expressed by osteoblasts and osteoclasts.

Thus, the reduced expression of MMP-9 associated with maintenance of histomorphometric and biomechanical parameters in the zinc-supplemented group suggests that zinc prevents bone resorption. In comparison, the high-level of MMP-9 expression in bone tissues of T1DM rats and reduced collagen content reinforce the association of MMPs with collagen in osteoporotic bone.

Furthermore, the increases in collagen density, trabecular spaces, Young’s modulus, and ultimate strain parameters and the reduced *MMP-9* gene expression in the T1DMS group compared to the T1DM group and the similarity of these values to the control group suggest that zinc supplementation protects against organic matrix degradation and bone loss.

The expression of *COL1A* in bone tissues of T1DMS rats was lower than that in T1DM rats, suggesting a positive effect on zinc on maintenance of bone metabolism through bone architecture (histomorphometric parameters) and bone strength and flexibility (biomechanical parameters) similar to the control group. Several studies have previously observed similar results regarding the protective effect of zinc supplementation against collagen degradation in *in vitro* studies [28,64], clinical studies in patients [55,65], and animal models [23,51].

*COL1A* expression in bone tissues of T1DM rats was upregulated, possibly indicating an increase in bone loss associated with a chronic diabetic condition. These results indicate high bone turnover as an alternative mechanism to maintenance of bone homeostasis that was not necessary for the T1DMS group, providing further support for the protective effect of zinc on the bone metabolism. Similar to our T1DM results, the *COL1A* response was also observed in *in vitro* studies, suggesting that hyperglycemia can affect bone tissue by inducing excessive production of osteoid matrix [17]. This observation may also be explained by the increase in *MMP-9* mRNA that possibly leads to degradation of the organic matrix in hyperglycemic conditions. Moreover, McCabe et al. [66] reported that the chronic exposure of osteoblasts to hyperglycemic cell culture media increased collagen production and altered the cellular phenotype toward that of osteocytes [67].

The discovery of RANK, RANKL, and OPG, factors involved in the control of osteoclast differentiation and osteoporosis, has advanced bone research into a new era. The RANK/RANKL/OPG system is an important signal transduction pathway that regulates bone resorption, modeling, and remodeling. The binding of OPG to RANKL inhibits binding between RANKL and RANK, thereby preventing osteoclast precursor differentiation and fusion to form mature osteoclasts. Thus, the relative concentrations of RANKL and OPG in bone are a major determinant of bone mass and strength.

*OPG* expression was downregulated in the T1DMS group, suggesting that bone homeostasis is maintained by zinc supplementation even in chronic hyperglycemic conditions. Moreover, the zinc effect on *OPG* mRNA expression is controversial. Iitsuka et al. [23] showed unaltered *OPG* mRNA expression during zinc supplementation for 14 days in a T1DM-induced bone loss animal model, and Fong et al. [68] reported similar *OPG* mRNA expression between postnatal and control groups. However, a review by Yamaguchi [22] showed that zinc plays an important role in bone growth by stimulating the expression of *OPG* mRNA in osteoblastic cells.

We observed upregulation of *OPG* in a chronic hyperglycemic condition, suggesting an attempted protective response against excessive bone loss induced by diabetic conditions. When *OPG* expression is increased relative to RANKL expression, the latter is expected to become unavailable to bind RANK in pre-osteoclasts, resulting in reduced bone resorption [17]. *OPG* upregulation in T1DM was also reported in T1DM patients in a previous study in our laboratory, indicating that diabetes during pubertal growth, which is associated with proinflammatory processes, may cause deficient bone-mass gain [8]. Moreover, an *in vitro* study has demonstrated excessive OPG synthesis at different glucose concentrations [17]. Thus, the upregulation of *OPG* expression in T1DM rats suggests that this increase protects the bone against resorption by inhibiting osteoclast differentiation mediated by RANK-RANKL binding. Although the present results show that the upregulation of OPG in T1DM rats did not result in detectable protection against trabecular structures, the loss of bone mass may have been more pronounced without this increased expression.

These results suggest an important role for zinc on bone protection in chronic T1DM and are supported by maintenance of bone architecture (histomorphometric and collagen content) and biomechanical proprieties and downregulation of genes involved in organic matrix degradation (*MMP-9* and *COL1A*).

Zinc supplementation also showed an anabolic effect, as evidenced by *OC* mRNA upregulation and increased serum ALP activity after the 90-day experimental period. *OC* mRNA is primarily expressed by post-proliferating and terminally mature osteoblasts [69–72] and regulates mineralization of the extracellular matrix [73–75]. ALP is an important serum marker associated with osteoblast activity and bone formation and is necessary for bone mineralization and development of collagenous structures [76]. Thus, the upregulation of *OC* and increased serum ALP activity are consistent with the hypothesis that zinc regulates osteoblastogenesis, suggesting a possible induction of bone formation and mineralization [22]. Furthermore, these results are consistent with Young’s modulus values showing a high resistance to fracture, supporting the suggestion that zinc supplementation may protect the bone architecture in both mineral and non-mineral content, leading to greater biomechanical strength.

In conclusion, zinc supplementation prevented bone loss in chronic T1DM rats as demonstrated by the maintenance of bone homeostasis and bone architecture, strength, and flexibility. In addition, zinc-induced *OC* upregulation and *RANKL*, *OPG*, *COL1A*, and *MMP-9* downregulation, even in chronic hyperglycemia, support a protective role for zinc in long-term diabetic conditions through stimulating expression of the mineralizing phenotype in osteoblasts and reducing expression of the resorptive phenotype in osteoclasts. Moreover, the protective zinc effect is supported after chronic hyperglycemia in T1DM leads to bone loss, as evidenced by alterations in bone structures associated with poor bone quality.

Thus, these results suggest the therapeutic potential of zinc supplementation as a complementary therapy to prevent bone loss in patients with diabetes or other related chronic diseases, resulting in better bone protection during growth as well as in adult and advanced ages.
